# Modelling cholera transmission dynamics in the presence of limited resources

**DOI:** 10.1186/s13104-019-4504-9

**Published:** 2019-08-01

**Authors:** Farai Nyabadza, Jennifer Mawunyo Aduamah, Josiah Mushanyu

**Affiliations:** 1 African Institute for Mathematical Sciences, (AIMS), Mbour, Senegal; 2 Department of Pure and Applied Mathematics, University of Johannesburg, Auckland Park, 2006 South Africa; 3 Department of Mathematics, University of Zimbabwe, Box MP 167, Mount Pleasant, Harare Zimbabwe

**Keywords:** Cholera, Nonlinear recovery rate, Hospital bed, Backward bifurcation, Basic reproduction number

## Abstract

**Objectives:**

We study the transmission dynamics of cholera in the presence of limited resources, a common feature of the developing world. The model is used to gain insight into the impact of available resources of the health care system on the spread and control of the disease. A deterministic model that includes a nonlinear recovery rate is formulated and rigorously analyzed. Limited treatment is described by inclusion of a special treatment function. Center manifold theory is used to show that the model exhibits the phenomenon of backward bifurcation. Matlab has been used to carry out numerical simulations to support theoretical findings.

**Results:**

The model analysis shows that the disease free steady state is locally stable when the threshold ${\mathcal {R}}_{0} < 1$. It is also shown that the model has multiple equilibria and the model exhibits the phenomenon of backward bifurcation whose implications to cholera infection are discussed. The results are useful for the public health planning in resource allocation for the control of cholera transmission.

## Introduction

Cholera is an acute gastro-intestinal infection and water-borne disease which is caused by the bacterium Vibrio Cholerae, *V. cholerae* $\text {O}1$ or $\text {O}139$. Vomiting and diarrhoea are its major characteristics and when patients are treated with delay, it can lead to severe dehydration and death within few hours. The disease has two modes of transmission: direct and the indirect transmission. Direct transmission (human–human) is very uncommon as compared to the indirect (environment–human) which occurs by ingesting contaminated food or water [1, 2]. An estimated 100,000–120,000 deaths are due to cholera every year in the world with only a small proportion being reported to World Health Organization (WHO) [3].

Cholera remains a significant threat to public health in the developing world, with cyclic outbreaks occurring twice per year in endemic areas [4]. For instance, more recently on the 6th of September 2018, a cholera outbreak in Harare was declared by the Ministry of Health and Child Care (MoHCC) of Zimbabwe [5]. As of 15 September 2018, 3621 cumulative suspected cases, including 71 confirmed cases, and 32 deaths had been reported (case fatality ratio: $0.8 \%$); of these, $98\%$ (3564 cases) were reported from the densely populated capital Harare [5]. The City of Harare is facing a plethora of challenges, notably insufficient safe water supplies, frequent sewer pipe bursts, uncollected refuse and rampant illegal vending [6]. This has negatively impacted on public health in the city exposing residents to diarrhoeal disease outbreaks, an upsurge in typhoid fever cases and sporadic outbreaks of cholera [6]. As of the year 2018, the disease has also claimed more than 67 lives in Zambia and Malawi did report some cases of cholera in Lilongwe. Thus, the cholera tragedy continues to devastate disadvantaged countries and communities. For more information about cholera and its occurrence, we refer the reader to [7, 8].

Several mathematical models describing cholera dynamics have been proposed and analyzed; see for instance [8–17]. These models differ from each other in some aspects. In most of these models, the recovery rate is assumed to be a constant. However, in reality the recovery rate depends on time of recovering process, which can be related to the number of infectious individuals seeking treatment and the basic factor; availability of health resources to the public. The resources of the health system includes the number of health care workers (physicians, nurses, pharmacists, etc.), capacity of the hospital settings (number of hospital beds and medicines) and the effectiveness and efficiency of the treatment. In many developing countries, resources of treatment are extremely scarce. So this approximation cannot reflect the real cure rate.

Motivated by the recent cholera outbreak in Zimbabwe which has overwhelmed the resources of the health care system, we formulate a compartmental deterministic mathematical model with a suitable treatment function in order to study the impact of limited hospital resource capacity on Cholera disease. The number of available hospital beds per 10,000 (hospital bed-population ratio) is used by health planners as a method of estimating resource availability to the public [18, 19]. Due to the significance of hospital bed-population ratio (HBPR), we will formulate the recovery rate incorporating the impact of limited resource capacity of the health care system in terms of HBPR for this work.

The paper is arranged as follows; in “Main text” section, we formulate and establish the basic properties of the model. The model is analysed for stability in “Analysis of the model” section. In “Numerical simulations” section, we carry out some numerical simulations. Parameter estimation and numerical results are also presented in this section. The paper is concluded in “Conclusions” section.

## Main text

### The model

The cholera model classifies the human population at time *t*, denoted by *N*(*t*), into susceptible individuals *S*(*t*), cholera infected individuals *I*(*t*) and recovered individuals *R*(*t*) such that,

$$\begin{aligned} N\left( t\right) = S\left( t \right) + I\left( t \right) + R\left( t \right) . \end{aligned}$$

An additional compartment *B*(*t*), representing the concentration of vibrios in contaminated water has also been incorporated in the model. We use the model in [8] and incorporate aspects of limited resources as proposed in [20]. The model involves assumptions which are of critical importance and these are:

i. The recovery rate depends on both the number of infectious individuals (*I*) and the hospital bed-population ratio (*b*).
ii. All the infected individuals cannot recover unless they get treated in hospitals.
iii. Recovered individuals are not permanently immune to the disease.

Susceptible individuals are recruited into the community either by birth or immigration at a rate $\mu N$. Susceptible individuals can be infected either through human-to-human transmission or by ingesting environmental vibrios from contaminated aquatic reservoirs at the rates $\beta _{1}I$ and $\beta _{2}\dfrac{B}{B + k}$, respectively. The recovery rate of infected individuals is given by $\gamma$. This recovery rate includes the hospital bed-population ratio, $b>0$ and also depends on infected individuals *I*, such that, it is a function of both *b* and *I*. The recovery rate $\gamma (b, I)$ is thus given as follows:

1 $$\begin{aligned} \gamma (b, I) = \gamma _{0} + (\gamma _{1} - \gamma _{0}) \frac{b}{I+b}, \end{aligned}$$

where $\gamma _{1}$ is the maximum per capita recovery rate due to the sufficient health care resource and few infectious individuals as well as the inherent property of a specific disease, $\gamma _{0}$ is the minimum per capita recovery rate due to the function of basic clinical resources. This recovery function was firstly used in [21]. The following assumptions for the recovery rate $\gamma (b, I)$ are made:

(*H*1): $$\gamma (b, I) > 0$$ for $$I \ge 0$$, $$b > 0$$, and $$\gamma (b, 0) = \gamma _{1} > 0$$,
(*H*2): $$\frac{\partial \gamma (b, I)}{\partial I} < 0$$, $$\displaystyle \lim _{I\rightarrow \infty } \gamma (b, I) = \gamma _{0} > 0$$ and $$\displaystyle \lim _{I\rightarrow \ 0} \gamma (b, I) = \gamma (b,0) = \gamma _{1}$$,
(*H*3): $$\frac{\partial \gamma (b, I)}{\partial b} > 0$$, $$\displaystyle \lim _{b\rightarrow \infty } \gamma (b, I ) = \gamma _{1}$$ and $$\displaystyle \lim _{b\rightarrow \ 0} \gamma (b, I ) = \gamma _{0}$$.

We assume a constant size population with natural death rate given by $\mu$. Cholera-infected individuals contribute to *V. cholerae* in the aquatic environment at rate $\alpha$ and vibrios have a net death rate $\delta$ in the environment. The differential equations for the Cholera model are;

2 $$\begin{aligned} \left\{ \begin{array}{l} \dfrac{dS}{dt} = \mu N - \beta _{1}\frac{SI}{N} - \beta _{2} \dfrac{SB}{B + k} - \mu S, \\ \dfrac{dI}{dt} = \beta _{1}\frac{SI}{N} + \beta _{2} \dfrac{SB}{B + k} - \gamma (b,I)I - \mu I, \\ \dfrac{dR}{dt} = \gamma (b,I)I - \mu R,\\ \dfrac{dB}{dt} = \alpha I - \delta B. \end{array} \right. \end{aligned}$$

We assume that all parameters are positive and the initial conditions of system (2) are given by: $S(0) = S_{0} > 0,$ $I(0) = I_{0} \ge 0,$ $R(0) = R_{0} \ge 0,$ $B(0) = B_{0} > 0.$

### Analysis of the model

#### Non-dimensionalization of the model

Our system of equations has different dimensions with respect to the human population and *V. cholerae*. To make system (2) dimensionless, the following substitutions are made: $S = sN$, $I = iN$, $R = rN$, $B=xN$, $k={\hat{k}}N$ and $b={\hat{b}}N$ with $s + i + r = 1$. The new system becomes:

3 $$\begin{aligned} \left\{ \begin{aligned} \dfrac{ds}{dt} \,&= \ \mu - \beta _{1}si - \beta _{2} \dfrac{sx}{x + {\hat{k}}} - \mu s, \\ \dfrac{di}{dt} \,&= \ \beta _{1}si + \beta _{2} \dfrac{sx}{x + {\hat{k}}} - \left[ \gamma _{0} + \left( \gamma _{1} - \gamma _{0} \right) \dfrac{{\hat{b}}}{i + {\hat{b}}} \right] i - \mu i, \\ \dfrac{dr}{dt} \,&= \ \left[ \gamma _{0} + \left( \gamma _{1} - \gamma _{0} \right) \dfrac{{\hat{b}}}{i + {\hat{b}}} \right] i - \mu r,\\ \dfrac{dx}{dt} \,&= \alpha i - \delta x. \end{aligned} \right. \end{aligned}$$

#### Positivity of solutions

Since system (3) describes changes in the population of humans, it is considered mathematically and epidemiologically well-posed if it satisfies the positivity and boundedness conditions.

##### **Lemma 1**

*Given that the initial conditions of system* (3) *are positive, the solutions* *s*(*t*), *i*(*t*), *r*(*t*) *and* *x*(*t*) *are non-negative for all* $t> 0$.

##### *Proof*

Assume that $t_{1} = \sup \{ t> 0: s> 0, i> 0, x > 0 \} \in (0, t]$. Thus, $t_{1} > 0$. Let $\lambda (t) = \beta _{1} i + \dfrac{\beta _{2} x}{x + {\hat{k}}}$, it follows from the first equation of (3) that,

$$\begin{aligned} s(t_{1}) \,\, \text {exp} \left\{ \,\, \mu t_{1} + \int _{0}^{t_{1}} \lambda (x) dx \right\} - s(0) \ge \int _{0}^{t_{1}} \mu \,\, \text {exp} \left\{ \mu y + \int _{0}^{y} \lambda (x) dx \right\} dy, \end{aligned}$$

so that,

$$\begin{aligned} s(t) & = s(0) \,\, \text {exp} \left\{ - \left( \mu t_{1} + \int _{0}^{t_{1}} \lambda (x) dx \right) \right\} \\&\times \left[ \text {exp} \left\{ -\left( \mu t_{1} + \int _{0}^{t_{1}} \lambda (x) \,dx\right) \right\} \right] \int _{0}^{t_{1}} \mu \,\, \text {exp} \left\{ \mu y + \int _{0}^{y} \lambda (x) \,dx\right\} \,dy > 0. \end{aligned}$$

Similarly, it can be shown that $i(t) > 0$, $r(t)>0$ and $x(t) > 0$, for all time $t > 0$. $\square$

#### Invariant region

##### **Theorem 1**

*Let* $\left( s(t), i(t), r(t), x(t) \right)$ *be the solution of system* (3) *with initial conditions* $( s_{0}, i_{0}, r_{0}, x_{0} )$. *The compact set,*

$$\begin{aligned} \Phi = \left\{ (s, i, r, x) \in {\mathbb {R}}^{4}_{+}, W_{H} \le 1, W_{B} \le \frac{\alpha }{\delta } \right\} \end{aligned}$$

*is positively invariant and attracts all solutions in* ${\mathbb {R}}^{4}_{+}$.

##### *Proof*

We follow the proof given in [22]. Consider, $W(t) = (W_{H}, W_{B}) = (s + i+r, x)$. The time derivative of *W*(*t*) is given by

$$\begin{aligned} \frac{dW}{dt}&= \left( \frac{W_{H}}{dt}, \frac{W_{B}}{dt} \right) = \left( \frac{ds}{dt}+\frac{di}{dt}+\frac{dr}{dt},~\frac{dx}{dt}\right) ,\\&= \left( \mu - \mu W_H , \quad \alpha i - \delta x \right) . \end{aligned}$$

This gives

4 $$\begin{aligned} {\left\{ \begin{array}{ll} \dfrac{dW_{H}}{dt} = \mu - \mu W_{H} \le 0, \quad \quad \text{ for } \quad W_{H} \ge 1,\\ \dfrac{dW_{B}}{dt} = \alpha i - \delta x \le \alpha W_{H} - \delta W_{B} \le 0, \quad \text{ for } \quad W_{B} \ge \dfrac{\alpha }{\delta }\\ \text{ with }~~W_H\ge 1~~\text{ and }~~\delta >0. \end{array}\right. } \end{aligned}$$

From (4), we have $\dfrac{dW}{dt} \le 0$ which implies that $\Phi$ is a positively invariant set. We also note that by solving (4) we have;

$$\begin{aligned} 0 \le \left( W_{H} (t), W_{B} (t) \right) \le \left( 1 + W_{H} (0) e^{-\mu t}, \frac{\alpha }{\delta } + W_{B} (0) e^{-\delta t} \right) , \end{aligned}$$

where $W_{H} (0)$ and $W_{B} (0)$ are the initial conditions of $W_{H} (t)$ and $W_{B}(t)$ respectively. Thus, $0 \le \left( W_{H} (t), W_{B}(t) \right) \le \left( 1, \dfrac{\alpha }{\delta } \right)$ as $t\rightarrow \infty$ and hence $\Phi$ is an attractive set. $\square$

#### Disease free steady state and the basic reproduction number

System (3) has a disease free steady state given by

$$\begin{aligned} {\mathcal {E}}_{0} = \left( s^{0}, i^{0}, r^{0}, x^{0} \right) = \left( 1 , 0, 0, 0 \right) , \end{aligned}$$

a scenario depicting an infection-free state in the community or society. The basic reproduction number, ${\mathcal {R}}_{0}$, defined as the expected number of secondary cases produced by a single infectious individual in a completely susceptible population over the duration of its infectious period, is a threshold parameter that allows us to predict whether the disease will die out or persist [23]. Generally, ${\mathcal {R}}_{0} < 1$ means that the disease cannot invade the population and ${\mathcal {R}}_{0} > 1$ means that each infected individual produces more than one secondary infected individual.

Denote the basic reproduction number of system (3) by

5 $$\begin{aligned} {\mathcal {R}}_0={\mathcal {R}}_i+{\mathcal {R}}_x~~~\text{ where }~~{\mathcal {R}}_i=\frac{\beta _1}{\mu +\gamma _1}~~\text{ and }~~{\mathcal {R}}_x=\frac{\alpha \beta _2}{{\hat{k}}\delta (\mu +\gamma _1)}. \end{aligned}$$

Here, ${\mathcal {R}}_0$ is the sum of two sub-reproduction numbers representing the contributions of individuals in compartments *i* and *x* respectively.

#### Local stability of the disease-free steady state

We now show that the disease-free equilibrium point ${\mathcal {E}}_0$ is locally asymptotically stable whenever ${\mathcal {R}}_0<1$.

##### **Theorem 2**

*The disease-free equilibrium point* ${\mathcal {E}}_0$ *of system* (3) *is locally asymptotically stable if* ${\mathcal {R}}_0< 1$ *and is unstable if* ${\mathcal {R}}_0>1$.

##### *Proof*

The Jacobian matrix of system (3) at ${\mathcal {E}}_0$ is given by

$$\begin{aligned} J({\mathcal {E}}_0)=\begin{bmatrix} -\mu&-\beta _1&0&-\frac{\beta _2}{{\hat{k}}}\\ 0&\beta _1-(\mu +\gamma _1)&0&\frac{\beta _2}{{\hat{k}}}\\ 0&\gamma _1&-\mu&0\\ 0&\alpha&0&-\delta \end{bmatrix}. \end{aligned}$$

We determine the local stability of the disease-free equilibrium by the following submatrix of $J({\mathcal {E}}_0)$,

$$\begin{aligned} J_1({\mathcal {E}}_0)=\begin{bmatrix} \beta _1-(\mu +\gamma _1)&0&\frac{\beta _2}{{\hat{k}}}\\ \gamma _1&-\mu&0\\ \alpha&0&-\delta \end{bmatrix}. \end{aligned}$$

We note that all off-diagonal elements are positive, thus we now consider matrix $-J_1({\mathcal {E}}_0)$. We claim that $-J_1({\mathcal {E}}_0)$ is an *M*-matrix. Multiplying matrix $-J_1({\mathcal {E}}_0)$ by the positive $3\times 1$ matrix $X_1=\left[ \mu \delta ,~\delta \gamma _1,~\alpha \mu \right] ^{T}$, we have

$$\begin{aligned} -J_1({\mathcal {E}}_0)\cdot X_1=(1-{\mathcal {R}}_0)\cdot X_2 \end{aligned}$$

where $X_2$ is a positive $3\times 1$ matrix given by $X_2=\left[ \mu \delta (\mu +\gamma _1),~0,~0\right] ^{T}$. Since $-J_1({\mathcal {E}}_0)$ is an *M*-matrix, it follows that all eigenvalues of $J_1({\mathcal {E}}_0)$ have negative real parts, which implies the local asymptotic stability of the disease-free equilibrium if ${\mathcal {R}}_0<1$. Also, we show that the determinant of $J_1({\mathcal {E}}_0)$ is given by

$$\begin{aligned} \text{ det }~J_1({\mathcal {E}}_0)=\mu \delta (\mu +\gamma _1)({\mathcal {R}}_0-1). \end{aligned}$$

Thus, the matrix $J_1({\mathcal {E}}_0)$ has eigenvalues with negative real parts if ${\mathcal {R}}_0<1$, which implies the stability of the disease-free equilibrium. This completes the proof. $\square$

#### Endemic steady state

The endemic equilibrium of system (3) always satisfies

6 $$\begin{aligned} \left\{ \begin{aligned}&\mu - \beta _{1}s^*i^* - \beta _{2} \dfrac{s^*x^*}{x^* + {\hat{k}}} - \mu s^* =0 , \\&\beta _{1}s^*i^* + \beta _{2} \dfrac{s^*x^*}{x^* + {\hat{k}}} - \left[ \gamma _{0} + \left( \gamma _{1} - \gamma _{0} \right) \dfrac{{\hat{b}}}{i^* + {\hat{b}}} \right] i^* - \mu i^*=0, \\&\left[ \gamma _{0} + \left( \gamma _{1} - \gamma _{0} \right) \dfrac{{\hat{b}}}{i^* + {\hat{b}}} \right] i^* - \mu r^*=0,\\&\alpha i^* - \delta x^*=0. \end{aligned} \right. \end{aligned}$$

From the third and last equation of (6), we have that

7 $$\begin{aligned} r^*=\frac{(i^*\gamma _0 +{\hat{b}}\gamma _1)i^*}{\mu (i^*+{\hat{b}})}~~~\text{ and }~~~x^*=\frac{\alpha i^*}{\delta }. \end{aligned}$$

Substituting the expression for $x^*$ in (7) into the first equation of (6) we obtain

8 $$\begin{aligned} s^*=\frac{\mu (\alpha i^*+\delta {\hat{k}})}{\alpha \beta _1 i^{*2}+\alpha \beta _2 i^*+\alpha i^* \mu +\beta _1 \delta i^*{\hat{k}}+\delta {\hat{k}} \mu }. \end{aligned}$$

Substituting (7) and (8) into the second equation of (6) leads to the following fourth order polynomial equation

9 $$\begin{aligned} i^*\left( \nu _{3} {i^{*}}^{3} + \nu _{2} {i^{*}}^{2} + \nu _{1} i^{*} + \nu _{0}\right) = 0. \end{aligned}$$

Solving (9) gives $i^*=0$ which corresponds to the disease-free equilibrium or

10 $$\begin{aligned} \nu _{3} {i^{*}}^{3} + \nu _{2} {i^{*}}^{2} + \nu _{1} i^{*} + \nu _{0} = 0, \end{aligned}$$

where

$$\begin{aligned} \left\{ \begin{aligned} \nu _{0} \,&=\ \mu \delta {\hat{b}}{\hat{k}}(\mu +\gamma _1)(1-{\mathcal {R}}_0) , \\ \nu _{1}\,&= \ \alpha {\hat{b}} \gamma _1 (\beta _2+\mu )+\mu (\alpha ({\hat{b}}-1) \beta _2+\alpha {\hat{b}} \mu +\gamma _0 \delta {\hat{k}}+\delta {\hat{k}} \mu )+\beta _1(\mu (({\hat{b}}-1) \delta {\hat{k}}-\alpha {\hat{b}})+{\hat{b}} \gamma _1 \delta {\hat{k}}), \\ \nu _2 \,&= \ \alpha (\beta _2+\mu )(\gamma _0+\mu )+\beta _1(\alpha {\hat{b}} \gamma _1+\mu (\alpha ({\hat{b}}-1)+\delta {\hat{k}})+\gamma _0 \delta {\hat{k}}),\\ \nu _3 \,&= \ \alpha \beta _1 (\mu +\gamma _0). \end{aligned} \right. \end{aligned}$$

We can clearly note that, $\nu _0>0\Leftrightarrow {\mathcal {R}}_0<1$ and $\nu _0<0\Leftrightarrow {\mathcal {R}}_0>1$. The number of possible positive real roots of polynomial (10) are determined using the Descartes Rule of Signs. The various possibilities for the roots are shown in the presentation below.

**Table:** 

	$$\nu _{3} > 0$$							
	$\nu _{2} > 0$				$\nu _{2} < 0$			
	$\nu _{1} > 0$		$\nu _{1} < 0$		$\nu _{1} > 0$		$\nu _{1} < 0$	
	$\nu _{0} > 0$	$\nu _{0} < 0$	$\nu _{0} > 0$	$\nu _{0} < 0$	$\nu _{0} > 0$	$\nu _{0} < 0$	$\nu _{0} > 0$	$\nu _{0} < 0$
	$(R_{0} < 1)$	$(R_{0} > 1)$	$(R_{0} < 1)$	$(R_{0} > 1)$	$(R_{0} < 1)$	$(R_{0} > 1)$	$(R_{0} < 1)$	$(R_{0} > 1)$
$i^{*}$	0	1	2	1	2	3	3	1

#### Existence of backward bifurcation

We establish conditions for the existence of backward bifurcation following Theorem 4.1 proven in [24]. We shall make the following change of variables: $s=x_{1},~i=x_2,~r=x_3,~x=x_4$, so that $\text{ N }=\displaystyle \sum\nolimits _{n=1}^{4}{x_n}$. We now use the vector notation $X=(x_{1},x_{2},x_{3},x_{4})^{T}$. Then, system (3) can be written in the form $\dfrac{dX}{dt}=F(t,x(t))=(f_{1},f_{2},f_{3},f_{4})^T$, where

11 $$\begin{aligned} \left\{ \begin{array}{l} \dfrac{dx_1}{dt} = \mu - \beta _{1}x_1x_2 - \eta \beta _{1} \dfrac{x_1x_4}{x_4 + {\hat{k}}} - \mu x_1 =f_1, \\ \dfrac{dx_2}{dt} = \beta _{1}x_1x_2 + \eta \beta _{1} \dfrac{x_1x_4}{x_4 + {\hat{k}}} - \left[ \gamma _{0} + \left( \gamma _{1} - \gamma _{0} \right) \dfrac{{\hat{b}}}{x_2 + {\hat{b}}} \right] x_2 - \mu x_2 =f_2, \\ \dfrac{dx_3}{dt} = \left[ \gamma _{0} + \left( \gamma _{1} - \gamma _{0} \right) \dfrac{{\hat{b}}}{x_2 + {\hat{b}}} \right] x_2 - \mu x_3=f_3,\\ \dfrac{dx_4}{dt} = \alpha x_2 - \delta x_4. \end{array} \right. \end{aligned}$$

Here $\beta _2=\eta \beta _1$, with the following possibilities on the value of $\eta$; $\eta =1$, $\eta \in (0,1)$ or $\eta >1$. Let $\beta _1$ be the bifurcation parameter, ${\mathcal {R}}_0=1$ corresponds to

12 $$\begin{aligned} \beta _1=\beta ^*_1=\frac{\delta {\hat{k}} \left( \gamma _1+\mu \right) }{\alpha \eta +\delta {\hat{k}}}. \end{aligned}$$

The Jacobian matrix of system (3) at ${\mathcal {E}}_0$ when $\beta _1=\beta ^*_1$ is given by

$$\begin{aligned} J^*({\mathcal {E}}_0)=\left( \begin{array}{cccc} -\mu &{} -\beta ^* _1 &{} 0 &{} -\frac{\eta \beta ^* _1}{{\hat{k}}} \\ 0 &{} -\mu +\beta ^* _1-\gamma _1 &{} 0 &{} \frac{\eta \beta ^* _1}{{\hat{k}}} \\ 0 &{} \gamma _1 &{} -\mu &{} 0 \\ 0 &{} \alpha &{} 0 &{} -\delta \\ \end{array} \right) \end{aligned}$$

System (11), with $\beta _1=\beta ^*_1$ has a simple eigenvalue, hence the center manifold theory can be used to analyze the dynamics of system (3) near $\beta _1=\beta ^*_1$. It can be shown that $J^*({\mathcal {E}}_0)$, has a right eigenvector given by $w=(w_1,w_2,w_3,w_4)^{T}$, where

13 $$\begin{aligned} w_1=-\delta \left( \gamma _1+\mu \right) ,~~w_2=\mu \delta ,~~w_3=\delta \gamma _1,~~w_4=\mu \alpha . \end{aligned}$$

Further, the left eigenvector of $J^*({\mathcal {E}}_0)$, associated with the zero eigenvalue at $\beta _1=\beta ^*_1$ is given by $v=(v_1,v_2,v_3,v_4)^{T}$, where

14 $$\begin{aligned} v_1=v_3=0,~~v_2=\alpha \eta +\delta {\hat{k}},~~v_4=\eta \left( \gamma _1+\mu \right) . \end{aligned}$$

We compute **a** and **b** in order to apply Theorem 4.1 in [24]. For system (11), the associated non-zero partial derivatives of *F* at the disease-free equilibrium are as follows:

$$\begin{aligned} \frac{\partial ^2 f_1}{\partial x_1\partial x_2} & = \frac{\partial ^2 f_1}{\partial x_2\partial x_1}=-\beta ^*_1,~~~~\frac{\partial ^2 f_1}{\partial x_1\partial x_4}=\frac{\partial ^2 f_1}{\partial x_4\partial x_1}=\frac{-\eta \beta ^*_1}{{\hat{k}}},~~~\frac{\partial ^2 f_1}{\partial x^2_4}=\frac{2\eta \beta ^*_1}{{\hat{k}}^2},\\ \frac{\partial ^2 f_2}{\partial x_1\partial x_2} & = \frac{\partial ^2 f_2}{\partial x_2\partial x_1}=\beta ^*_1,~~~~\frac{\partial ^2 f_2}{\partial x_1\partial x_4}=\frac{\partial ^2 f_2}{\partial x_4\partial x_1}=\frac{\eta \beta ^*_1}{{\hat{k}}},~~~\frac{\partial ^2 f_2}{\partial x^2_2}=\frac{2(\gamma _1-\gamma _0)}{{\hat{b}}},\\ \frac{\partial ^2 f_2}{\partial x^2_4} & = -\frac{2\eta \beta ^*_1}{{\hat{k}}^2},~~~~\frac{\partial ^2 f_3}{\partial x^2_2}=-\frac{2(\gamma _1 -\gamma _0)}{{\hat{b}}},~~~\frac{\partial ^2 f_1}{\partial x_2\partial \beta ^*_1}=-1,~~~\frac{\partial ^2 f_1}{\partial x_4\partial \beta ^*_1}=\frac{-\eta }{{\hat{k}}},\\ \frac{\partial ^2 f_2}{\partial x_2\partial \beta ^*_1} & = 1,~~~\frac{\partial ^2 f_2}{\partial x_4\partial \beta ^*_1}=\frac{\eta }{{\hat{k}}}. \end{aligned}$$

It thus follows that

$$\begin{aligned} {\mathbf{a }} & = v_2w_1w_2\frac{\partial ^2 f_2}{\partial x_1\partial x_2}+v_2w_1w_4\frac{\partial ^2 f_2}{\partial x_1\partial x_4}+v_2w^2_2\frac{\partial ^2 f_2}{\partial x^2_2}+v_2w^2_4\frac{\partial ^2 f_2}{\partial x^2_4}\\ & = \frac{\mu \theta _2 (\alpha \eta +\delta {\hat{k}})}{{\hat{b}}{\hat{k}}^2}\left( {\hat{b}}^*-{\hat{b}}\right) ,~~~~\left( {\hat{b}}^*=\frac{\theta _1}{\theta _2}\right) , \end{aligned}$$

with

$$\begin{aligned} \theta _1 =2 \left( \gamma _1-\gamma _0\right) \delta ^2 {\hat{k}}^2\mu>0~~\text{ and }~~\theta _2 =\beta ^* _1 \left( 2 \alpha ^2 \eta \mu +\gamma _1 \delta ^2 {\hat{k}}^2+\delta ^2 {\hat{k}}^2 \mu +\alpha \gamma _1 \delta \eta {\hat{k}} +\alpha \delta \eta {\hat{k}} \mu \right) >0. \end{aligned}$$

Note that if ${\hat{b}} <{\hat{b}}^*$ then $\text{ a }>0$ and $\text{ a }<0$ if ${\hat{b}}>{\hat{b}}^*$. Lastly,

$$\begin{aligned} {\mathbf{b }}=\frac{\mu (\alpha \eta +\delta {\hat{k}})^2}{{\hat{k}}}>0. \end{aligned}$$

We thus have the following result

##### **Theorem 3**

*If* ${\hat{b}} <{\hat{b}}^*$, *then system* (3) *has a backward bifurcation at* ${\mathcal {R}}_0=1$.

**Fig. 1:**
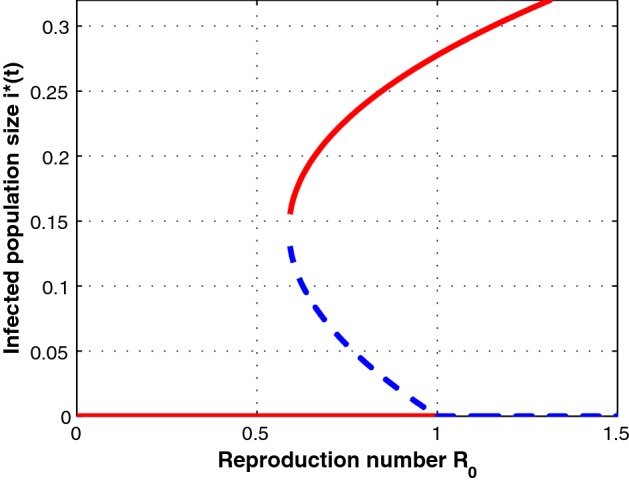
The figure showing a backward bifurcation. The *solid lines* denote stable states and the *dotted lines* denote unstable states

We observe from the results above that a backward bifurcation occurs at ${\mathcal {R}}_0 = 1$ if and only if ${\hat{b}}<{\hat{b}}^*$ is satisfied. From this, we can deduce that when the hospital bed-population ratio, ${\hat{b}}$ is below the critical threshold ${\hat{b}}^*$, then the number of hospital beds available to the population are below capacity and thereby lead to some individuals failing to access treatment. In such a case the prevalence of cholera infection remains high leading to a backward bifurcation, see Fig. 1. The existence of a backward bifurcation is also illustrated through numerical example by creating bifurcation diagram around ${\mathcal {R}}_0 =1$ (Fig. 1). To draw a bifurcation curve (the graph of $i^*$ as a function of ${\mathcal {R}}_0$), we fix $\mu = 0.03;~\beta _1 = 0.2;~\beta _2 = 0.1;~{\hat{k}} = 0.9;~{\hat{b}} = 0.1;~\gamma _0 = 0.006;~\gamma _1 = 0.13;~\alpha = 0.282;~\delta = 0.5$. For this case we have that ${\hat{b}}^*=0.1676 >{\hat{b}}$. The *solid lines* denote stable states and the *dotted lines* denote unstable states.

##### *Remark*

When the model exhibits backward bifurcation, reducing ${\mathcal {R}}_0$ below unit is not sufficient to control the cholera epidemic.

### Results and discussion

### Numerical simulations

We perform some numerical simulations of system (3) to support our theoretical findings.

#### Estimation of parameters

Parameter values used for numerical simulations are given in Table 1.

**Table 1:** Parameter values used in numerical simulations

Parameters	Description	Range	Sample value	Unit	References
$\mu$	Natural death rate of humans	$5.00 e^{-2}$–$6.00 e^{-2}$	0.00524/0.06	day$^{-1}$	Assumed
$\beta _{1}$	Effective contact rate between individuals	0.057–0.100	0.060/0.107	day$^{-1}$	Assumed
$\beta _{2}$	Per capita contact rate for humans and the contaminated environment	0.2073–0.2213	0.2143/0.22	day$^{-1}$	[9]
$${\hat{k}}$$	Half-saturation constant	$$10^{5}$$–$$10^{9}$$	$$10^{5}{/}10^{6} $$	Cells L$$^{-1}$$	[8]
$\gamma _{0}$	Minimum recovery rate of human	–	$(0.015, \dots )$	-	Assumed
$\gamma _{1}$	Maximum recovery rate of human	–	$(\gamma _{0} , 0.09)$	–	Assumed
${\hat{b}}$	Hospital bed-population ratio	–	(0, 20)	–	[20]
$\delta$	Bacterial net death rate	–	30	day$^{-1}$	[17]
$\alpha$	Shedding rate	1–150	50	Cells mL$^{-1}$ person$^{-1}$ day$^{-1}$	[8, 9]

#### Numerical results

Using the parameter values from Table 1, we obtain $$R_{ 0} < 1$$ for the initial conditions $$s(0) = 0.80$$, $$ i(0) = 0.15$$, $$r(0) = 0.05$$, $$ x(0) = 0.40$$.

**Fig. 2:**
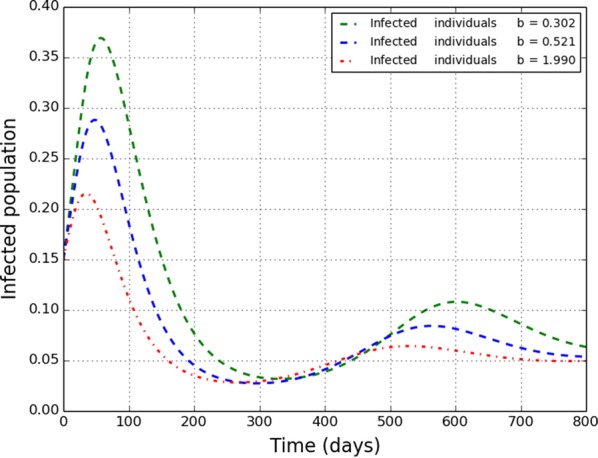
Varying limited resources *b* for $R_{0} > 1$

The limited resource parameter *b*, is varied in Fig. 2. It is shown that as *b* is increased, the infection population decrease. This means that increasing *b*, that is, when hospital-beds are increased during an outbreak, there is a high chance that the disease will not persist.

## Conclusions

A deterministic compartmental model with a nonlinear recovery rate was formulated to study and analyze the impact of available resources of the health care system on the transmission dynamics of Cholera. The recovery rate accounts for the number of available hospital beds per 10,000 population represented by the parameter ${\hat{b}}$ which is the critical index reflecting the resources of the health care system available to the public. Compared with previous cholera models, the work contained in this study is the first attempt to model the impact of limited resources of the health care system on the spread of cholera, with particular emphasis on the hospital beds.

It was shown that the disease free steady state is locally asymptotically stable whenever ${\mathcal {R}}_{0}<1$ and unstable otherwise. Inclusion of a non linear recovery rate has resulted in the existence of multiple endemic equilibria and the model exhibiting the phenomenon of backward bifurcation. The classical ${\mathcal {R}}_0$-threshold is not the key to control disease spread within a population. This was shown to result, in particular when the parameter ${\hat{b}}$ is low enough below ${\hat{b}}^*$. However, the cases of cholera infection decrease if there are a sufficient number of hospital beds, that is, when ${\hat{b}}>{\hat{b}}^*$. Therefore, in order to eradicate the disease in a community, effort must be targeted to increasing hospital resources.

## Limitations

Like in any model development, the model is not without limitations. The model can be extended by inclusion of other control measures such as vaccination and disinfection.

## Data Availability

Estimation of parameters have been stated throughout the body of the paper and included in the reference section. The graphs were produced using the MATLAB software that is available from https://www.mathworks.com/products/matlab.html.

## Abbreviations

WHO: World Health Organization
MoHCC: Ministry of Health and Child Care
HBPR: hospital bed-population ratio
